# Retrospective Identification of a Broad IgG Repertoire Differentiating Patients With *S. aureus* Skin and Soft Tissue Infections From Controls

**DOI:** 10.3389/fimmu.2019.00114

**Published:** 2019-02-07

**Authors:** Fabio Rigat, Erika Bartolini, Mattia Dalsass, Neha Kumar, Sara Marchi, Pietro Speziale, Domenico Maione, Luqiu Chen, Maria Rosaria Romano, Maria-Luisa Alegre, Fabio Bagnoli, Robert S. Daum, Michael Z. David

**Affiliations:** ^1^GSK Pharmaceuticals R&D, Stevenage, United Kingdom; ^2^GSK Vaccines R&D, Siena, Italy; ^3^Department of Pediatrics, University of Chicago, Chicago, IL, United States; ^4^Department of Engineering, University of Pavia, Pavia, Italy; ^5^Biochemistry Section, Department of Molecular Medicine, University of Pavia, Pavia, Italy; ^6^Department of Medicine, University of Chicago, Chicago, IL, United States; ^7^Department of Medicine, University of Pennsylvania, Philadelphia, PA, United States

**Keywords:** *Staphylococcus aureus*, skin infection, antibodies, microarray, antibody response

## Abstract

**Background:** Although the relevance of humoral immunity for protection against *S. aureus* skin and soft tissue infections (SSTIs) has been suggested by several animal and human studies, the question of which human antibodies may be protective has so far impeded the development of a safe and effective vaccine. Because most adults have developed certain anti-*S. aureus* antibodies due to *S. aureus* colonization or infection, we hypothesized that the titers of antibodies to *S. aureus* in uninfected controls would differ from those in infected patients and would also differ in infected patients from the time of acute infection to a 40-day convalescent serum.

**Methods:** To test these hypotheses, we measured human antibody levels against a panel of 134 unique antigens comprising the *S. aureus* surfome and secretome in subjects with active culture-confirmed *S. aureus* SSTIs (cases) and in controls with no infection, using a novel *S. aureus* protein microarray.

**Results:** Most *S. aureus* SSTI patients (*n* = 60) and controls (*n* = 142) had antibodies to many of the tested *S. aureus* antigens. Univariate analysis showed statistically weak differences in the IgG levels to some antigens in the SSTI patient (case) sera compared with controls. Antibody levels to most tested antigens did not increase comparing acute with 40-day serum. Multiple logistic regression identified a rich subset of antigens that, by their antibody levels, together correctly differentiated all cases from all controls.

**Conclusions:** Antibodies directed against *S. aureus* antigens were present both in patients with *S. aureus* SSTIs and in uninfected control patients. We found that SSTI patients and controls could be distinguished only based on differences in antibody levels to many staphylococcal surface and secreted antigens. Our results demonstrate that in the studied population, the levels of anti-*S. aureus* antibodies appear largely fixed, suggesting that there may be some level of unresponsiveness to natural infection.

## Introduction

*Staphylococcus aureus* is a common cause of human infections, particularly of skin and soft tissue infections (SSTIs) (1). Resistance has developed to all classes of antimicrobial agents introduced to treat this species. Methicillin-resistant *S. aureus* (MRSA) strains, resistant to nearly all β-lactam drugs, have caused several waves of resistance since 1960 (2), first in the health care setting and then, beginning in the 1990s, in the community (3). A vaccine may be the most effective intervention to reduce the incidence of infections caused by this dangerous pathogen. However, many individuals can be infected repeatedly, even by a single clone of *S. aureus* (4), demonstrating that in at least some cases, immune responses to natural *S. aureus* infection itself are not necessarily protective. Attempts to develop an effective vaccine for *S. aureus* have not succeeded to date (5), and there is growing evidence that T cell responses, and not only opsonophagocytic antibodies, play an important role in protective immunity (6–15). Vaccine development has also been hampered by *S. aureus* being a commensal organism, with recent studies suggesting that the prevalence of carriage of this organism in certain populations is >50% (16). Some groups have detected anti-*S. aureus* antibodies in healthy controls without a history of recent or current SSTI (17–20), suggesting that colonization alone may result in the development of a broad repertoire of anti-staphylococcal antibodies. However, it is not clear whether the specificity and titers of these antibodies can be distinguished from the antibodies generated during or immediately following an acute *S. aureus* skin infection.

Unlike many bacterial pathogens, the ability of *S. aureus* to evade the adaptive and innate host immune response in cases of furunculosis or other recurrent infections led investigators to undertake several studies of the molecular mechanisms of bacterial immune evasion (21–24). Nonetheless, the role of antibodies in protection from infection is not established.

Recently, a study demonstrated the feasibility of an investigation of antibody responses against many *S. aureus* antigens in a case-control study comparing uninfected controls to samples taken from *S. aureus* ST239 bacteremia patients during acute infection and again seven days later. They found higher antibody levels to a small set of antigens in the bacteremia patients compared to controls (25). Using a similar case-control study design, we set out to determine if there were differences in the *S. aureus* surfome and secretome antibody repertoire in the serum of patients with acute, uncomplicated culture-confirmed *S. aureus* SSTIs, the same patients 40 days later, and a group of matched control patients with no active infection.

## Materials and Methods

### Human Samples

We enrolled subjects with SSTIs at the University of Chicago Pediatric and Adult Emergency Departments in October 2009–July 2012. In addition, for each enrolled case with an SSTI, we enrolled 2 control subjects matched by age, gender and ethnicity who were treated in the same Emergency Department for a complaint that was not infectious, as previously described (16, 26). For all subjects, demographic information was collected. Among controls, 74.3% presented with a minor trauma, 9.7% with headache, 4.9% with chest pain, 3.5% with an eye-related complaint, and 7.6% had other complaints.

Blood samples were obtained from cases at enrollment and after 40 days, hereafter referred to as day 0 (D0) and day 40 (D40), and from controls at the single time point when they presented for care. Whole blood was centrifuged and serum collected, aliquoted and stored at −20°C for later analysis. Cultures were obtained to assess *S. aureus* colonization at 3 body sites (nares, oropharynx, and inguinal or perirectal region) at D0 and D40 for cases and at enrollment for controls. All *S. aureus* isolates obtained from infections and colonization cultures underwent genotyping as previously described (16). This study was approved by the Institutional Review Board of the Biological Sciences Division of the University of Chicago and was conducted in accordance with the Declaration of Helsinki. Written informed consent was obtained from each of the subjects.

### Staphylococcal Protein Microarray Design

A protein array of recombinant *S. aureus* antigens was generated as previously described (27) with a few modifications. For complete methods, see the Supplementary Methods. Briefly, the *S. aureus* proteins selected to be printed on the arrays represented surface and secreted factors identified *in silico* using a combined bioinformatics approach (9, 28). In particular, this list included 134 antigens, belonging to the *S. aureus* strain NCTC 8325 or Newman, produced in *E. coli* as recombinant His6- or GST-tagged proteins (Supplementary Table S3); and 2 capsular polysaccharides type 5 (CP5) and type 8 (CP8) isolated and purified from *S. aureus* type 5 or type 8 strains; and lipoteichoic acid (LTA; SIGMA Catalog number ti tlrl-slta, *Invivo*, USA). Protein microarrays containing a total of 134 staphylococcal antigens were generated. The microarray underwent validation experiments to confirm the efficiency of the protein deposition on the chips, as detailed in the (Supplementary Materials).

### Serological Profiling by Microarray Analysis

Sera obtained from SSTI (case) subjects and controls were analyzed in a blind-random fashion. They were tested for their immunologic reactivity with the panel of surface or secreted staphylococcal antigens in the microarray, which allows high throughput analysis of sera against a large number of antigens. Sera diluted 1:1,000 were evaluated by detecting total IgG bound to each protein spot using fluorescently labeled anti-human IgG and measuring the mean fluorescence intensity (MFI) values for each antigen, as detailed in the (Supplemental Materials).

### Overview of Analysis Approach

First, we tested the null hypothesis that the anti-*S. aureus* serum antibody titers against each antigen were not different between cases and controls and between case samples measured at the two tested time points. To prevent confounding of the interpretation of case-control comparisons due to differences in the cases in which *S. aureus* was cultured and those in which *S. aureus* was not cultured, only the IgG levels measured in the 30 culture-confirmed *S. aureus* cases were compared with those of the controls in our primary analyses. In many non-*S. aureus* infection cases, no culture was sent from SSTI patients. Second, we investigated whether the antibody titers against a subset of antigens could together discriminate cases from controls using penalized multiple logistic regression.

### Statistical Methods

The two-sample *t*-test and the Chi-square test for proportions were applied to assess differences in demographic characteristics and in *S. aureus* colonization comparing cases with controls.

The protein array MFI data were first analyzed using hierarchical clustering for descriptive purposes. These MFI data were then mapped onto calibrated anti-human IgG titers, averaged across technical replicates and background-filtered (methods in Supplementary Materials). These derived data are hereafter referred to as IgG levels.

Two-way analysis of variance (ANOVA) was applied to the log-IgG levels to assess the statistical significance of the observed average differences across antigens and subjects.

The IgG levels against each antigen measured in the SSTI patients in acute (D0) and late (D40) phase were compared to each other using the Wilcoxon test statistic. D0 and D40 data were separately compared to the IgG levels measured in the controls against each antigen using the Mann-Whitney test statistic. Penalized logistic regression (29–31) was used to establish whether the IgG levels against several antigens could correctly identify the infection status of all samples. Statistical significance of the identified antigen subset was established by comparing its accuracy, sensitivity and specificity to their null values derived respectively from the random classifier and by random mismatching of the measured IgG levels from their infection status. All calculations were performed using standard numerical routines implemented in the statistical package R version 3.3.3 (https://cran.r-project.org/).

## Results

### Study Population

Antibody responses against all 134 *S. aureus* surface or secreted antigens were generated for a total of 60 SSTI subject blood samples collected both at D0 and at D40 and for 142 controls, for a case:control ratio of ~1:2 and total sample size of 202 subjects.

### Demographic Characteristics of All Cases and Controls Are Similar

Table 1 shows the demographic characteristics of all 202 subjects. Consistent with the findings of Kumar et al. (16), we found no evidence that age, gender or race were significantly different in cases and controls (all *p*-values >> 0.05).

**Table 1:** Demographic characteristics of the analyzed case (*n* = 60) and control (*n* = 142) subjects.

**Characteristic**	**Cases, *N* = 60**	**Controls, *N* = 142**
**Age (years)**, Median (range)	34 (21-61)	32 (19-77)
**GENDER**		
Male	28 (47%)	66 (44%)
Female	32 (53%)	76 (56%)
**RACE**		
African American	59 (98%)	131 (92%)
Other	1 (2%)	9 (8%)

### *S. aureus* Colonization Prevalence Was Lower in Controls Than in Case Patients

Table 2 shows the results of the laboratory cultures performed to ascertain *S. aureus* colonization in the 60 SSTI cases and 142 healthy controls at the time of emergency room visit. *S. aureus* colonization at one or more body sites among cases at D0 (44/60 = 73%) was greater than in the controls (80/142= 56%; *p*-value < 0.05), whereas colonization of D40 cases (32/60 = 47%) was not significantly different from the controls. Among the SSTI case subjects colonized by any *S. aureus* strain, 30% (13/44) at D0 and 28% (9/32) at D40 carried USA300 compared with 12.5% (10/80) of the colonized controls. Despite USA300 colonization prevalence being higher at both time points among the SSTI subjects compared with controls, statistical significance (*p*-value < 0.05) was achieved only for the D0 cases.

**Table 2:** *S. aureus* colonization status for controls and for SSTI cases at D0 and D40, all of whom were cultured at three different body sites and also *S. aureus* infection status for SSTI cases cultured at one or more SSTI site on D0.

**Infection status**		***S. aureus*** colonization status^*^, ***n*** **(%)**				***S. aureus*** **SSTI culture result^#^, ***n*** (%)**			
		**No *S. aureus***	***S. aureus***			**No *S. aureus***	***S. aureus***		
			**USA300 MRSA**	**Other strain types**	**Total**		**USA300 MRSA**	**Other strain types**	**Total**
**SSTI cases (*****N*** **= 60)**	**D0**	16 (26%)	13 (22%)	31 (52%)	44 (73%)	30 (50%)	21 (35%)	9 (15%)	30 (50%)
	**D40**	28 (47%)	9 (15%)	23 (38%)	32 (47%)		no infection sites cultured		
**Controls D0 (*****N*** **= 142)**		62 (44%)	10 (7%)	70 (49%)	80 (56%)		no infection sites cultured		

* Result of laboratory cultures collected to assess asymptomatic colonization at one or more body site

# *Result of laboratory cultures collected at one or more SSTI site*.

### Descriptive Analysis of Raw Protein Array Data

Agglomerative clustering was applied to the Pearson correlations between raw MFI protein array data measured from the 60 SSTI cases (Figure S3). The dendrogram on top of Figure S3 shows that the MFI data measured across all antigens in samples pertaining to the same subject were found more similar to each other compared to the MFIs measured from any other individual for 53/60 ≈88% subjects. Also, the darker bands across most columns in Figure S3 show that several antigens reacted with antibodies present in the sera of most subjects. These descriptive analyses suggested that the individual immune signatures did not exhibit marked changes over time and that antibody responses in most subjects varied considerably across antigen subsets.

### Background Filtering and Calibration of MFI Protein Array Data

To ensure that the protein array data could be analyzed accurately, we assessed which MFI data could be calibrated as a proxy to the antibody concentration. Raw data ranging from ~4,000 to 30,000 MFI exhibited a constant four-fold variability of their calibration curves across slides (see Supplementary Materials). Pronounced assay saturation ensued for MFIs >60,000, where the variability of the calibration curves was drastically reduced. None of the calibrated antigen responses for each of the 134 antigens was found to lie below the 95th percentile of the MFI background distribution, corresponding to ~2,000 MFIs or an IgG level of 0.0056 mg/ml (see Supplementary Materials).

### IgG Levels Varied Significantly Across *S. aureus* Antigens and Across Subjects

**Figure 1:**
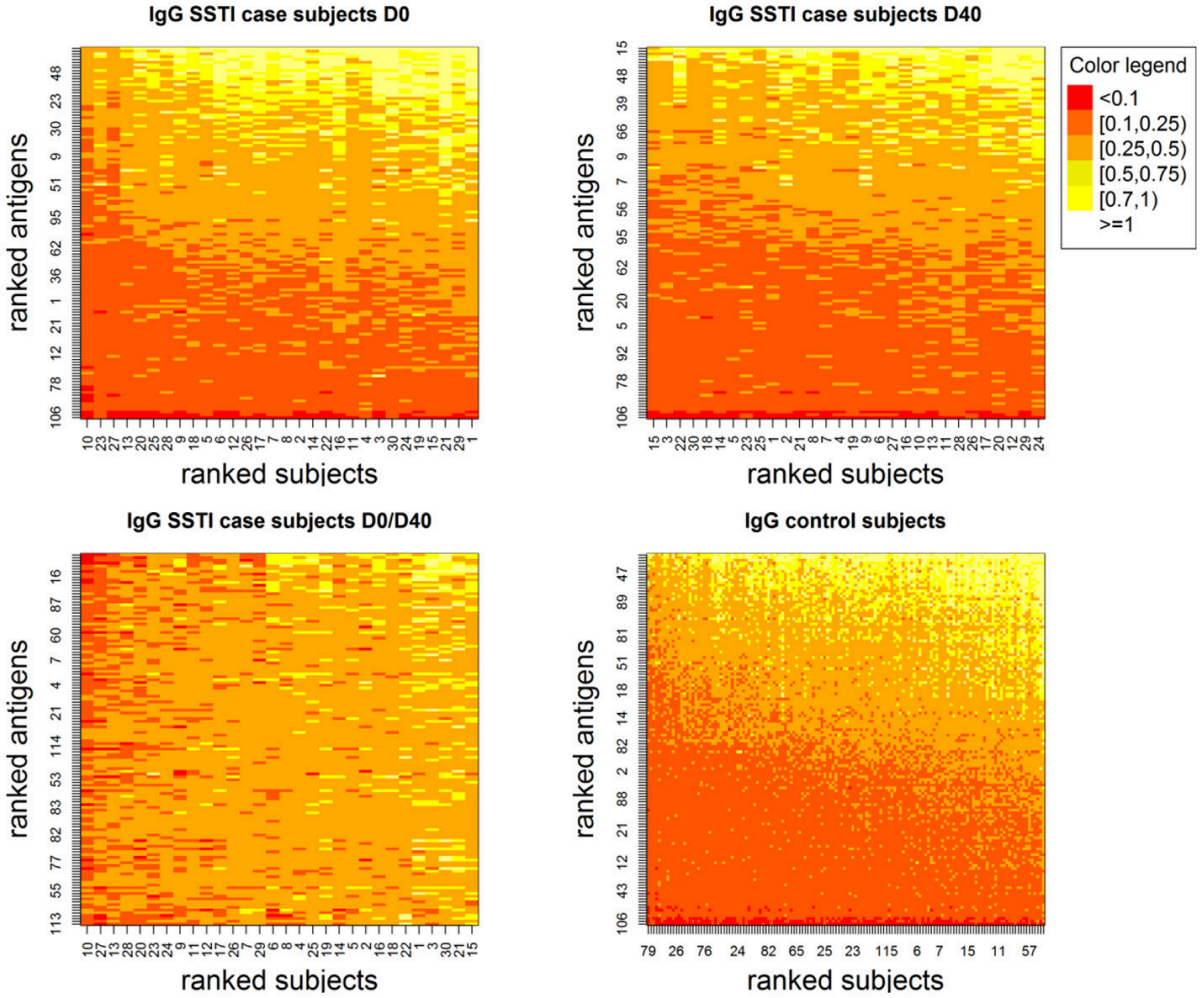
Showing IgG levels (anti-human IgG mg/ml) for each antigen (y-axis) derived through the calibration process for the SSTI cases at the two measured time points (**Top**) and for the control subjects (**Bottom Right**). Low values are shown in darker tones and higher values are shown in progressively lighter tones. Although few antigens are consistently associated with very low IgG levels, antibodies were detectable against most *S. aureus* antigens. Also, among case subjects, temporal changes in IgG were observed both across subjects and antigens (**Bottom Left**).

Figure 1 depicts the IgG levels for all 60 SSTI cases at D0 and D40 (top panels) and for the controls (bottom left panel). Within each panel, subjects (rows), and antigens (columns) are ranked by their median IgG levels starting from the lowest (bottom left, shown in red) up to the highest (top right, shown in white). IgG levels above the background level were detectable against most antigens.

The bottom right panel in Figure 1 also shows that temporal changes in IgG levels were observed comparing D0 and D40 both across SSTI subjects and *S. aureus* antigens.

**Figure 2:**
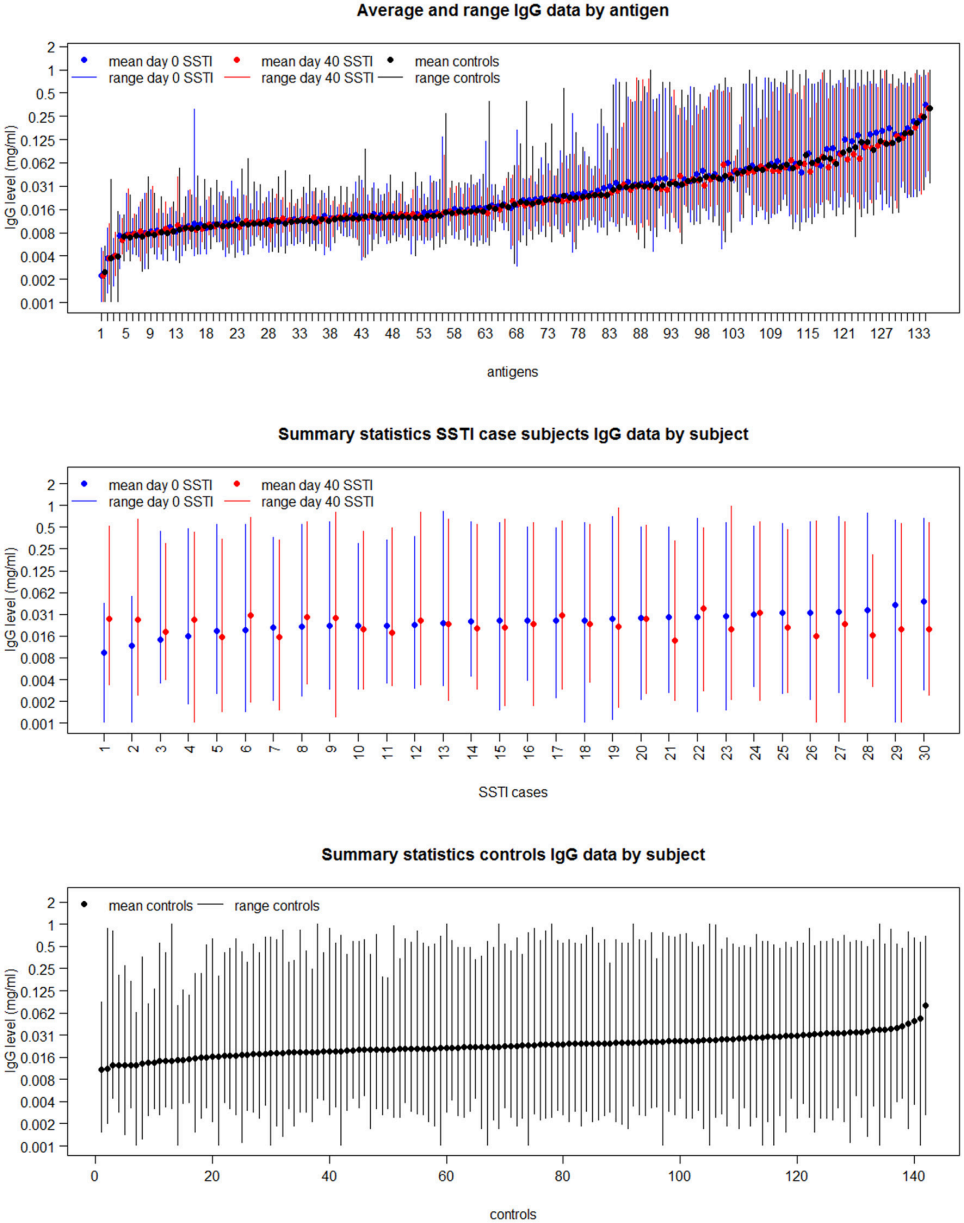
The mean and range of IgG levels for each antigen calculated across case and control subjects (**Top**); and the mean and range of the 134 IgG levels for each of the 30 culture-confirmed *S. aureus* SSTI case subject serum samples (**Middle**) and for each control subject serum sample (**Bottom**).

The top panel in Figure 2 shows the range (vertical bars) of the IgG levels for each antigen and their average (shown as dots) calculated across the SSTI D0 data (shown in blue), the SSTI D40 data (shown in red) and for the controls (shown in black). The descriptive summaries suggest that while antibody levels varied, there was little difference comparing cases to controls and comparing D0 and D40 data from cases. The middle and bottom panels in Figure 2 show the variation in range and the average of the IgG levels for each SSTI and control subject calculated across antigens. Two-way ANOVA was applied to the logarithms of D0, D40 and control IgG data consistently showing statistically significant changes across *S. aureus* antigens and across subjects (*R*^2^ > 92%, *p* < 0.001).

### IgG Levels in Confirmed *S. aureus* SSTI Cases Were Different From Those Measured in Other SSTI Cases

Table 3 shows the list of antigens associated with statistically significant univariate differences (*p*-values < 0.05) between the IgG levels measured in the 30 culture-confirmed *S. aureus* SSTIs and those measured in the 30 SSTIs not confirmed to be due to *S. aureus*. The IgG levels measured at D0 against each of these antigens in the *S. aureus* confirmed SSTI sera were greater than in the other SSTI serum samples, and they were all lower than those in other sera at D40. This result demonstrates that several *S. aureus*-specific immune responses were detectable among the SSTI cases even though the genetic backgrounds of infecting *S. aureus* isolates varied. To prevent confounding of the interpretation of case-control comparisons due to these differences, only the IgG levels measured in the 30 culture-confirmed *S. aureus* cases were compared with those of the controls in subsequent analyses.

**Table 3:** Percent differences between the median IgG levels among case subjects with culture-confirmed *S. aureus* SSTIs and those with SSTIs not confirmed to be due to *S. aureus* for the antigens with Mann-Whitney *p*-value < 0.05.

**Time point**	**Antigen ID**	**% difference median IgG *S. aureus* SSTIs—median IgG SSTIs not *S. aureus***	**Mann-Whitney *p*-value**
**D0**	SAOUHSC_00749	20	0.008
	HlgC	32	0.01
	NuC	15	0.01
	SAOUHSC_02887	30	0.02
	NWMN_1877	58	0.02
	SAOUHSC_01920	15	0.02
	SAOUHSC_00256	17	0.025
	SAOUHSC_02333	19	0.027
	SAOUHSC_00174	16	0.04
	SAOUHSC_00404	16	0.047
	SAOUHSC_02463	16	0.048
**D40**	SAOUHSC_00400	−57	0.005
	SAOUHSC_00427	−49	0.01
	CoA	−86	0.01
	ClfB	−26	0.02
	SAOUHSC_00671	−59	0.02
	SasF	−51	0.03
	SAOUHSC_00399	−32	0.03
	IsdA	−74	0.04

### SSTI Cases and Controls Had Different IgG Levels Against Several *S. aureus* Antigens

**Figure 3:**
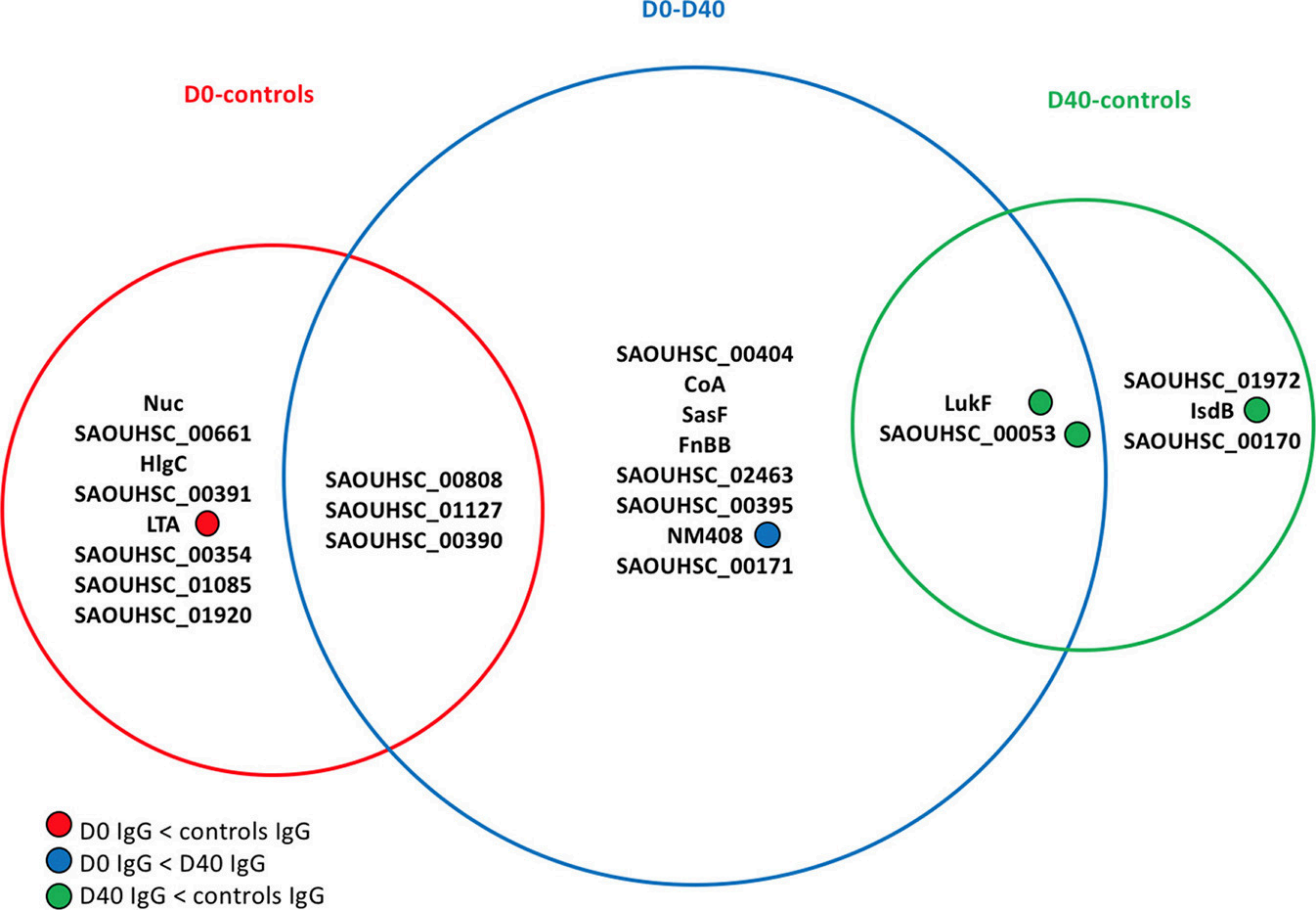
Venn diagram showing the antigens having statistically significant differences (*p*-value < 0.05) in analyses comparing the frequency distributions of the IgG levels measured in the 30 culture-confirmed *S. aureus* SSTI subject serum samples at D0 and D40 or between the IgG levels measured in the 30 SSTI serum samples and the samples from controls. The area of each circle is proportional to the number of antigens carrying significantly different IgG levels measured across subjects in each pair-wise comparison. A total of 11/134 (8%), 13/134 (10%), and 5/134 (4%) antigens carried different IgG levels for the D0-controls, D0-D40, and D40-controls pair-wise comparisons, respectively. For information about the antigens included here, (see the Supplementary Materials, Table S1).

Figure 3 lists the antigens associated with statistically significant univariate differences (*p*-value < 0.05) between the IgG levels of the 30 *S. aureus* SSTI cases at D0 and D40 or between the IgG levels of cases and controls. The area of each circle in Figure 3 is proportional to the number of antigens showing significantly different IgG levels, that is 11/134 (8%), 13/134 (10%), and 5/134 (4%) for the D0-controls, D0-D40, and D40-controls pair-wise comparisons, respectively. The *p*-values associated with these tests ranged between 0.004 and 0.046, showing weak evidence against the null hypothesis of no difference between the IgG levels against individual antigens between cases and controls at the usual 0.05 *p*-value threshold. If the more conservative *p*-value threshold 0.005 is applied (32), the antigens showing statistically significant differences are reduced to: SasF and SAOUHSC_00808 when comparing D0 to D40 IgG levels, *Nuc* when comparing D0 data to controls, and LukF and Csa1B for comparisons between D40 and controls (see Figure 3).

Only IgG levels against NM408 were significantly higher in the D40 case samples compared with D0, demonstrating that the humoral immune response against *S. aureus* antigens detectable in these samples was weak. LTA was associated with greater IgG levels in control sera compared to the D0 case sera. The IgG levels against 3 out of the 5 statistically significant antigens in the D40-controls comparison (LukF, Csa1B, IsdB) were greater in the controls compared with the D40 case (SSTI) serum samples.

For each dataset (day0 cases, day40 cases, controls) we assessed whether the measured serum reactivity within each group was detectably explained by gender, age, or race and found no association (data not shown).

### A Large Subset of Antibody Responses Against *S. aureus* Antigens When Combined Correctly Distinguish Between *S. aureus* Cases and Controls

**Figure 4:**
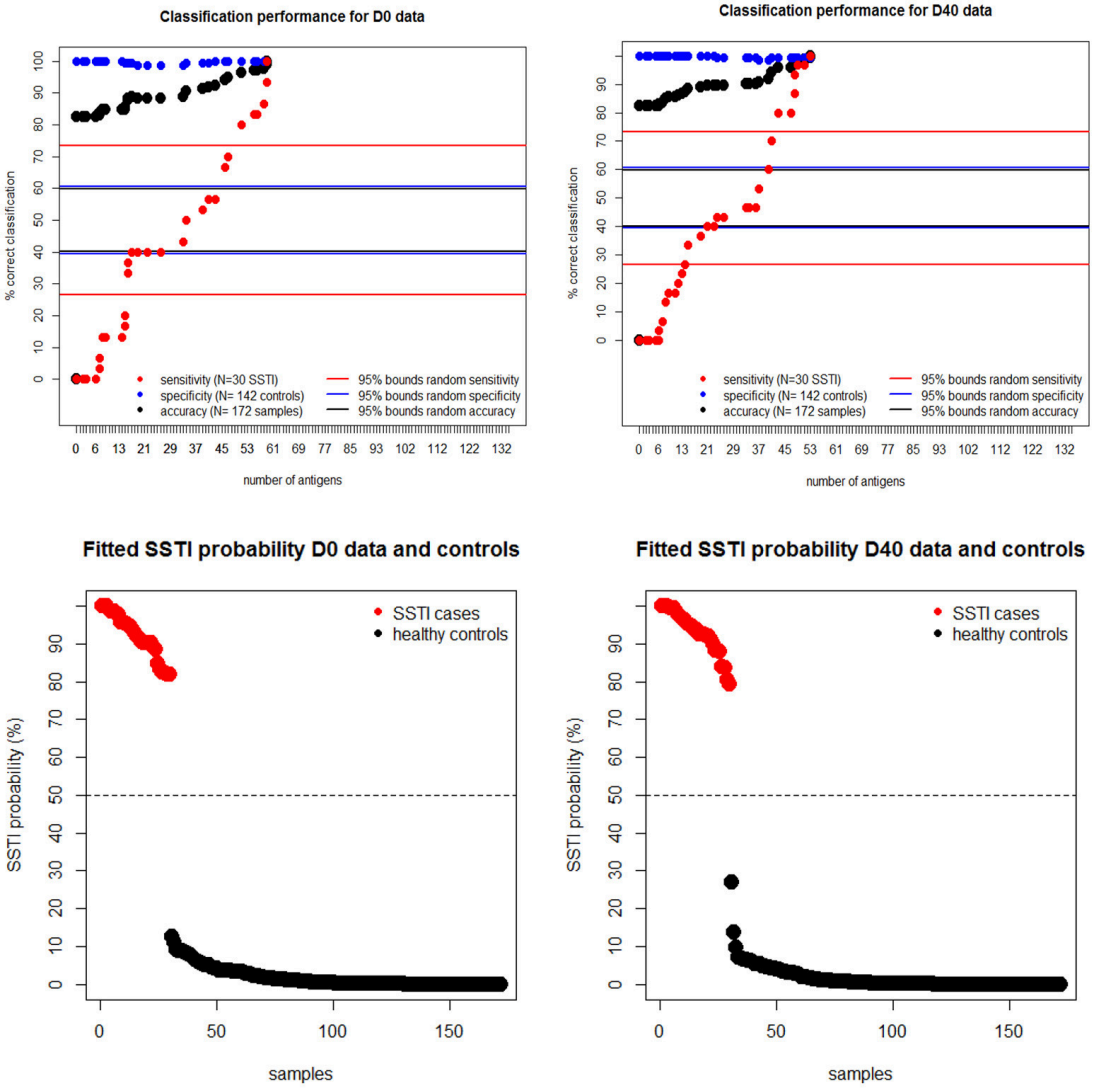
Upper panels: regression sensitivity (red dots) at D0 **(left)** and D40 **(Right)** improved monotonically in the size of the antigen set selected by the penalized multiple logistic regression while specificity (blue dots) was roughly constant. The horizontal lines show the 95% probability intervals of the random classifier (40–61% for accuracy in black, 27–73% for sensitivity in red and 40–62% for specificity in blue lines). Bottom panels: the smallest fitted *S. aureus* SSTI probability among SSTI cases was 78% and the highest SSTI probability among the controls was 29%, showing a clear separation of the fitted infection status on the basis of the measured IgG levels.

To estimate the protective potential of the immune responses directed against multiple *S. aureus* antigens, penalized multiple logistic regressions were fitted to the IgG levels. This statistical model allowed for the identification of the smallest set of antigens for which combined IgG levels would be able to distinguish all cases from controls. The upper panels in Figure 4 show that the proportion of correctly classified *S. aureus* SSTI cases (sensitivity) improved monotonically in the size of the set of antigens included in the model while the proportion of correctly classified controls (specificity) was roughly constant, so that the overall proportion of correctly classified subjects (accuracy) increased monotonically. This result showed that the expected propensity of the model to classify all subjects as controls, due to the high proportion (142/172 ≈83%) of control subjects informing this analysis, was overcome when the IgG levels against many antigens were included among the *S. aureus* SSTI subject predictors. The same results were observed when the *S. aureus* SSTI case antigen levels were weighted proportionally to the inverse of the case-control ratio. The horizontal lines in Figure 4 show that sensitivity exceeded the upper 95% probability bound of the random classifier (73%) when respectively 51 (D0 and controls) and 43 (D40 and controls) antigens were selected by the model, and if all subjects were correctly classified as SSTI cases or controls when respectively 59 (D0 and controls) and 53 (D40 and controls) antigens were selected. The bottom panels in Figure 4 demonstrate the separation of the *S. aureus* SSTI cases from controls based on the weighted average of the IgG levels fitted by the logistic regression.

**Figure 5:**
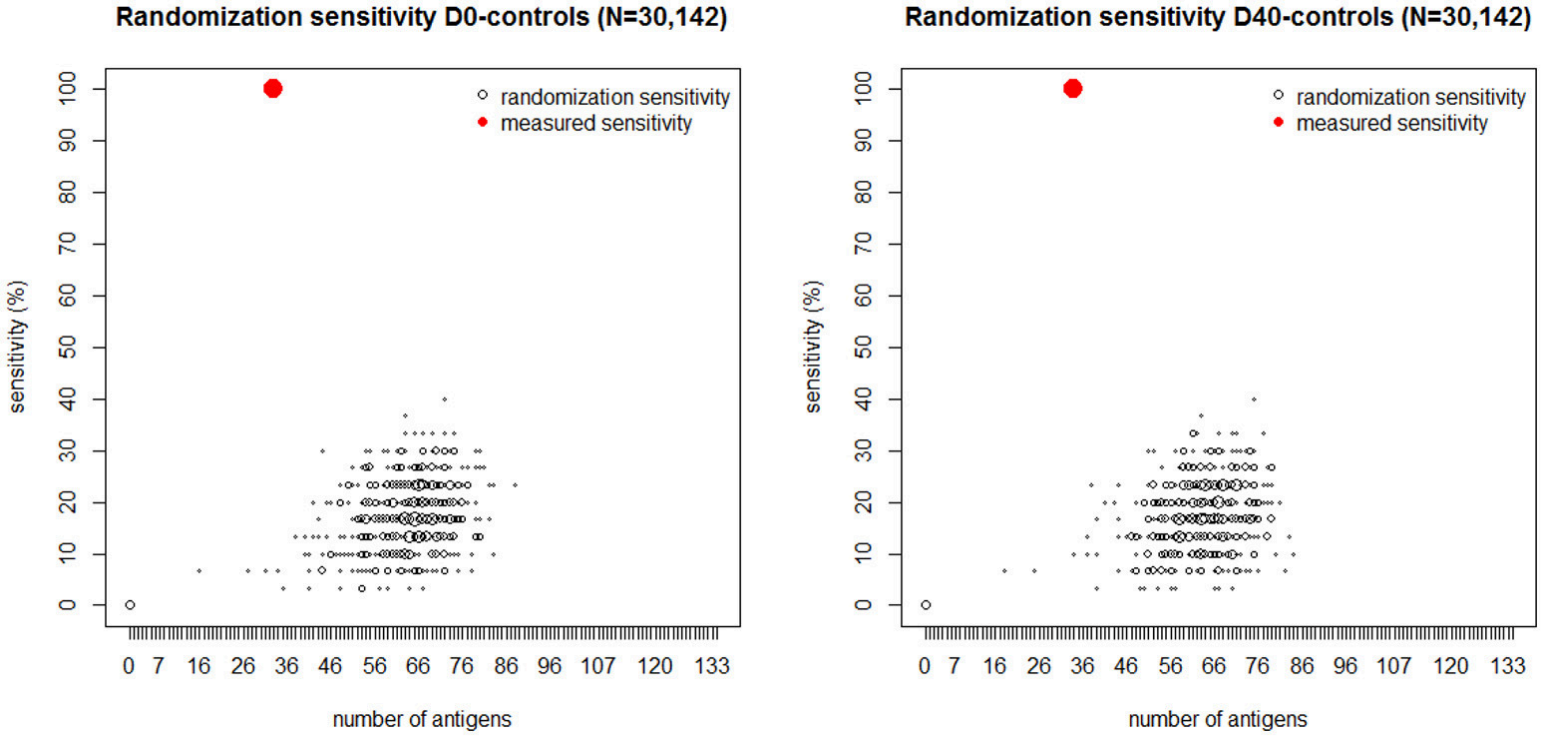
Black dots: number of antigens (horizontal axis) selected by the logistic regression plotted vs. its sensitivity (vertical axis) generated by randomly mismatching the measured IgG profiles measured at D0 (**Left**) and D40 (**Right**) and in the controls from their corresponding SSTI infection status. The size of each dot is proportional to the frequency of its coordinate values over 10,000 randomizations. The red dot in each figure panel shows the number of antigens and corresponding sensitivity obtained by fitting the logistic regression to the IgG data and their corresponding SSTI infection status. The absence of black dots near the red dots shows that the probability that 100% sensitivity could be achieved by chance is numerically zero. These results lead us to reject the null hypothesis that the association between SSTI infection status and IgG profiles is not statistically significant.

To further assess the statistical significance of these results, (Figure 5) depicts another analysis demonstrating the probability distribution of the classification sensitivity obtained by randomly mismatching the observed *S. aureus* SSTI infection status from its corresponding IgG levels. The size of each empty black circle in Figure 5 reflects its relative frequency over ten thousand iterative randomizations. In both cases the maximum classification sensitivity of 40% was attained using the randomly mismatched IgG levels of over 65 antigens. Antigens represented by red circles within each figure panel show the sensitivity value achieved using the observed D0-controls dataset (left) and D40-controls dataset (right) vs. the corresponding number of antigens. The pronounced gap between each set of antigens represented by black circles and those represented by the corresponding red circles in Figure 5 shows that the probability of correct classification of all SSTI samples by chance using the observed IgG levels is numerically zero.

**Figure 6:**
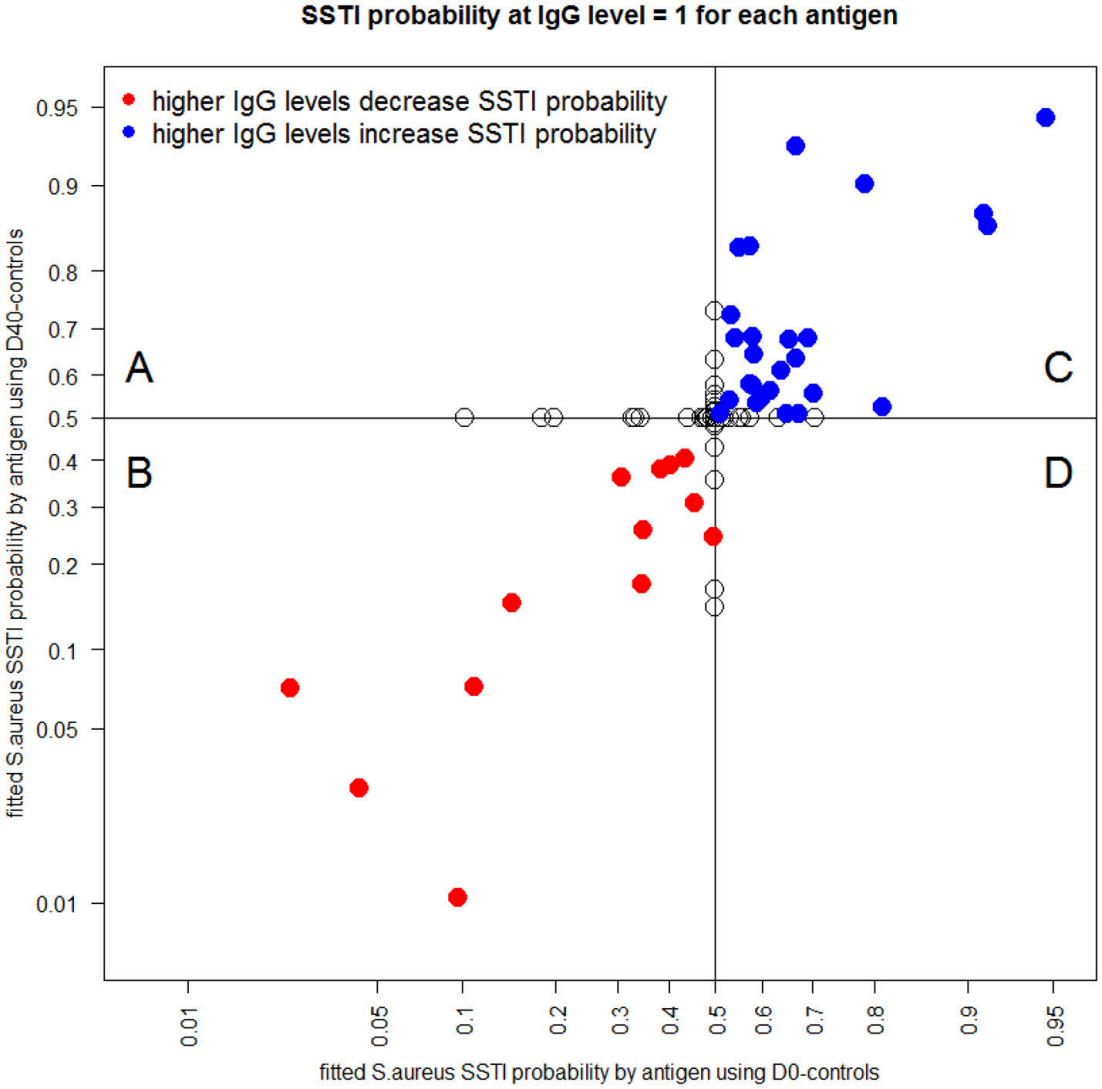
Each dot depicts the *S. aureus* SSTI classification probabilities for the D0-controls (horizontal axis) and the D40-controls (vertical axis) when IgG is set to the value 1 μg/mL for the corresponding antigen and IgG = 0 μg/mL for all other antigens. Red circles [quadrant **(B)**] mark antigens (see list in Table S1) which higher IgG levels were associated with a decrease in the SSTI classification probabilities both when comparing the D0 data and the D40 data to the controls. Blue circles [quadrant **(C)**] represent antigens which higher IgG levels were associated with an increase in the SSTI classification probabilities. Empty circles [quadrants **(A)** and **(D)**] identify antigens associated with antibody responses having no association with SSTI classification probability in one or both case-control comparisons.

Each point in Figure 6 shows the classification probability of an acute *S. aureus* SSTI serum sample (D0, plotted on the horizontal axis) and of a convalescent serum sample after a *S. aureus* SSTI (D40, on the vertical axis) when the IgG level against a single tested antigen was set to 1 mg/ml and the IgG levels against all remaining 133 antigens were set to zero. Red circles (Figure 6B and Table S2) mark the IgG levels against 13 antigens associated with low *S. aureus* SSTI probabilities. Blue circles (Figure 6C) mark the IgG levels against 26 antigens associated with low *S. aureus* SSTI probabilities estimated from the same case-control data. Points with zero coordinates on either axis in Figure 6 identify antigens for which IgG levels had no individual association with *S. aureus* SSTI in one or both case-control analyses. These results show that co-occurrence of high IgG levels against the 13 red-labeled antigens and low IgG levels against the 26 blue-labeled antigens minimized the probability that samples were classified as *S. aureus* SSTI in this population.

## Discussion

This study demonstrates that the protein microarray platform is an effective high-throughput method to analyze simultaneously many human antibody responses against a broad set of bacterial antigens. Univariate analysis of the IgG levels measured using this platform showed that both SSTI cases and controls had measurable serum antibodies against most of the 134 tested *S. aureus* surfome/secretome antigens and that the levels of these antibodies changed little in the context of and in the aftermath of a clinically significant *S. aureus* infection. To the best of our knowledge, this is the first study to document this phenomenon in a large group of *S. aureus* SSTI patients and in contemporaneously enrolled controls. Our findings suggest that generation of detectable anti-staphylococcal antibodies to surface and secreted antigens as a result of natural exposure to *S. aureus* is nearly universal in this population. These findings also suggest some level of unresponsiveness to natural infection for antibody levels that may reflect B cell-intrinsic or T cell-dependent responses. Alternatively, it is possible that anti-staphylococcal antibody levels are generally very high in this population and that an active infection cannot elicit additional antibody production. Also, these results suggest that it is unlikely that a single or a small number of IgG responses can be used to assess for recent or current non-invasive *S. aureus* SSTIs.

Multivariate case-control analyses showed that it is possible to combine the antibody responses to a large set of antigens to correctly classify patients with an acute *S. aureus* SSTI, distinguishing them from uninfected control patients (33). These findings provide a first step toward a quantitative systematic understanding of antibody-mediated protection against *S. aureus* SSTIs. In particular, if *S. aureus* SSTIs are preventable through selective development of antibodies, these findings support the hypothesis that protection is likely to require elicitation of an antibody response signature against many antigens. This hypothesis is consistent with the variety of antigens that were individually assessed in prior literature, which suggested that presence of antibodies to multiple *S. aureus* proteins correlates with protection from a more severe infection. For example, in one study of patients with *S. aureus* bacteremia, those with higher levels of antibodies to 64 *S. aureus* antigens were less likely to develop sepsis (34), suggesting a protective effect of antibodies in the case of bacteremia. Others found that bacteremia patients had a higher antibody level to certain *S. aureus* antigens compared with a control population (25), suggesting an antibody response to acute invasive infection. Also, in yet another cohort study, antibodies to two virulence factors, Panton Valentine leukocidin (PVL) and alpha toxin (Hla), were studied in children. Patients who suffered an invasive infection, compared with a non-invasive infection, had lower baseline antibody levels to Hla. Higher titers of anti-Hla antibodies correlated with a lower risk of recurrent infection (35). Dryla et al. (17) found that patients infected at a variety of anatomic sites, compared with uninfected controls, may have higher levels of certain antibodies to cell wall antigens of *S. aureus*, suggesting that active infection may result in antibody responses to these antigens. In another study, antibodies against toxic shock syndrome toxin (TSST) protected against toxic shock syndrome, a disease with a high mortality (36). Also, Adhikari et al. determined that among patients with *S. aureus* bacteremia, those with higher serum IgG levels to a number of *S. aureus* toxins and virulence factors (Panton-Valentine leukocidin [PVL], Hla, delta-hemolysin, staphylococcal enterotoxin C-1, and phenol-soluble modulin α-3) were less likely to progress to sepsis (37). In contrast, Hermos et al. found that there was no protection against *S. aureus* SSTI from high antibody levels against PVL, a toxin almost uniformly produced by CA-MRSA isolates (38).

Consistent with this literature, our findings suggest that a putative protective role of antibodies directed toward single *S. aureus* antigens reflects a complex relationship between antigenicity and immunity. A natural antibody response to any single antigen likely cannot protect a person against *S. aureus* SSTI. In fact, in the population that we studied in Chicago, most people had antibodies to most *S. aureus* surface and secreted antigens and even with these antibodies, they likely remained at risk of developing a *S. aureus* SSTI.

Despite the clear strength in terms of breadth of the analyzed set of antigens, there are limitations to our study. First, due to the limited sample size of this study the IgG levels that were found to distinguish cases and controls either individually or in combination may not accurately distinguish these groups in other populations and need further validation. Second, *S. aureus* genotype may vary among infecting isolates in our subjects, potentially making the group of culture-confirmed *S. aureus* SSTI case subjects heterogeneous. Third, we do not know if controls had had *S. aureus* infections in the past nor if cases had had *S. aureus* infections in the past, or if antibody responses to other commensal staphylococcal species overlap responses to *S. aureus* antigens. It is possible that antibody titers may have been affected by those prior, unobserved infections. Nevertheless, the nearly uniformly present array of anti-staphylococcal antibodies suggests that current infection status of a subject does not significantly affect the anti-*S. aureus* antibody repertoire directed against surface-expressed and excreted *S. aureus* antigens. Vaccination, however, may induce greater humoral and cellular immune responses to selected antigens than induced by a natural infection (39). Indeed, vaccine clinical trials have shown a significant response to vaccine antigens as compared with baseline levels (40, 41). The results of our study suggest that vaccine efficacy trials may have failed to date because the tested vaccines targeted only single antigens, and a combination vaccine, containing several antigens, may elicit a broader immune response than that induced by a natural infection. Further clinical research is thus needed to understand whether a specific signature of protective antibodies can be defined.

## Author Contributions

FB, RD, FR, MZD, and EB designed and conceived the study. EB and DM conceived and performed the protein microarray analysis. LC prepared the serum samples. SM and PS performed the SA antigen purification. MR CP5 and CP8 purification. FR and MD performed statistical analyses. MZD, FR, and EB drafted the manuscript. M-LA, DM, FB, RD, MZD, FR, and EB critically revised the manuscript. All authors approved the manuscript before it was submitted.

### Conflict of Interest Statement

FB, EB, SM, and DM, were employees of Novartis Vaccines at the time of the study. MD was a PhD student and collaborated with GSK at the time of the study as part of the PhD training. Following the acquisition of Novartis Vaccines by the GSK group of companies in March 2015, FR, EB, SM, DM, and FB are now employees of the GSK group of companies. FR, DM and FB own shares of the GSK group of companies. FB owns patents on *S. aureus* vaccine candidates. The remaining authors declare that the research was conducted in the absence of any commercial or financial relationships that could be construed as a potential conflict of interest.
