# Supplementary material for: Retrospective Identification of a Broad IgG Repertoire Differentiating Patients With S. aureus Skin and Soft Tissue Infections From Controls
Source: Front Immunol. 2019 Feb 7;10:114. doi: 10.3389/fimmu.2019.00114 (PMC6375365; doi:10.3389/fimmu.2019.00114)
Supplement: Supplementary file 1 [file Data_Sheet_1.docx]

**SUPPLEMENTARY MATERIALS**

**Isolation and purification of *S. aureus* type 5 and type 8 capsular polysaccharides**

*S. aureus* was grown in solid medium to provide a bacterial suspension of 600-800 mL. 30-50 g of wet cell pellet was collected by centrifugation at 8,000 rpm. The harvested pellet was washed three times with 50 mM Tris+2 mM MgSO_4_ pH 7.5, suspended in MilliQ water (0.5-0.6 g/mL) and stirred vigorously while the temperature was increased to 100ºC. Acetic acid was then added to a final concentration of 1% and the mixture was incubated at 100ºC for 2 hours. The mixture was neutralized with 1 M NaOH and centrifuged at 8,000 rpm. The supernatant was separated and added to 0.05 mg/ml of DNase/RNase (Sigma-Aldrich). The mixture was incubated for 5-7 hours at 37°C, and the supernatant was recovered by centrifugation at 8,000 rpm. 180 U/ml of mutanolysin (Sigma-Aldrich) was added to the suspension and the mixture incubated overnight (~16 hours) at 37°C with gentle stirring. The supernatant was recovered by centrifugation at 8,000 rpm and dia-filtered by tangential flow filtration (TFF) using a Tandem mod. 1082 Sartorius^TM^ system 5 and a 30 kDa cut-off membrane (Hydrosart Sartocon Slice 200, Sartorius), against 10 volumes of 1 M NaCl followed by 10 volumes of 10 mM sodium phosphate pH 7.2 buffer.

The dia-filtration retentate solution was further purified by anionic exchange chromatography, using a DEAE Sepharose^TM^ Fast Flow resin (G&E Healthcare) equilibrated with 10 mM sodium phosphate buffer pH 7.2. The sample was loaded at a concentration of 10 mM sodium phosphate pH 7.2, and 0-1 M NaCl gradient in 20 column volumes was applied for material elution. Chromatographic fractions were analyzed by ^1^H-NMR and those containing capsular polysaccharide were dia-filtered by TFF using a Tandem mod. 1082 Sartorius^TM^ system 5 and a 30 kDa cut-off membrane (Hydrosart Sartocon Slice 200, Sartorius), against 10 volumes of distilled water to remove residual salts. Purified polysaccharide was characterized for protein, nucleic acid, and peptidoglycan residual content.

**Characterization of *S. aureus* type 5 and type 8 capsular polysaccharides**

*Protein and nucleic acid content*

The protein concentration was estimated by MicroBCA, Lowery and Bradford protein assay kits, using bovine serum albumin (BSA) as a reference (Thermo Scientific).

Nucleic acid content was determined by UV spectroscopy at a wavelength of 260 nm.

*Identity and conformity*

The structure of collected polysaccharides was determined by ^1^H-NMR. Samples were dissolved in deuterium oxide (D_2_O, 99.9% atom D, Aldrich). ^1^H spectra were recorded by a Bruker Avance III 400 MHz at 25°C, using a 5-mm broadband probe (Bruker). The transmitter was set at the HDO frequency which was also used as a reference signal (4.79 ppm). Monodimensional proton NMR spectra were collected using a standard one-pulse experiment. The TOPSPIN 2.1 software package (Bruker) was used for data acquisition and processing of spectra.

*Peptidoglycan content*

Peptidoglycan content was quantified by amino acid analysis, performed using a High Performance Anionic Exchange Chromatography-Pulsed Amperometric Detection (HPAEC-PAD) method. Briefly, this analysis consisted of an acid hydrolysis *“in vacuum”* with 6 M HCl for 24 hours at 112°C, followed by chromatographic analysis using HPAEC-PAD equipped with AminoPac PA1 (Thermo) column and gradient elution in sodium acetate/NaOH. The chromatographic quantification was performed using a standard solution of amino acids in the concentration range 2.5-50 μM.

**Staphylococcal Protein Microarray construction, imaging and data analysis**

A protein array of recombinant *S. aureus* antigens was generated as previously described (Scietti L., et al 2016). Briefly*, S. aureus* antigens were expressed in *Escherichia coli* as His6- or GST-tagged recombinant proteins, purified from the cytoplasmic fraction as soluble forms as previously described (Bagnoli 2015; Scietti 2016; Roche 2003; O’Connell 1998). Recombinant antigens were spotted in 8 replicates on nitrocellulose-coated slides (FAST slides, Maine Manufacturing) using the no-contact Marathon Spotter (Arrayjet, Edinburgh, UK) resulting in spots of ~100 μm in diameter. A standard curve made of eight concentrations (twofold dilution from 0.5 to 0.004 mg/ml) of fluorescent Human IgG was used to assess comparison of replicate experiments.

Preliminary slide validation experiments, in which the slides were probed with mouse anti-GST and anti-6xHis monoclonal antibodies followed by detection with AlexaFluor®647-conjugated anti-mouse IgG secondary antibody (Jackson Immunoresearch), were performed to confirm the efficiency and reproducibility of the protein deposition on the chips. Nonspecific binding was minimized by preincubating protein or peptide microarray slides with a blocking solution (BlockIt, ArrayIt) for 1 hour. Sera were diluted 1:1000 in BlockIt buffer and overlaid on the protein and peptide arrays, respectively, for 1 h, at room temperature. AlexaFluor®647-conjugated anti-human IgG secondary antibody (Jackson Immunoresearch) was added for 1 h at room temperature in the dark, before proceeding with slide scanning. Fluorescence signals were detected using a confocal laser scanner (Power Scanner, Tecan Trading AG, Switzerland), and the 16-bit images were generated with PowerScanner software v1.2 at 10 μm/pixel resolution and processed using ImaGene 9.0 software (Biodiscovery Inc, CA). Elaboration and analysis of image raw fluorescence Intensity (FI) data were performed using in-house developed software and R scripts version 3.3.3 (https://cran.r-project.org/). For each serum sample the mean fluorescence intensity (MFI) of replicate spots was determined by subtraction of the background value surrounding each spot.

**Calibration, dimensionality reduction and background filtering of protein chip MFI raw data**

*Calibration*

Let *MFI_ijr_* be replicate *r=1,...,R* mean fluorescence intensity (MFI) measured from the serum of subject *i=1,...,I* against antigen *j=1,...,J*. These measurements were generated for all subjects at the fixed serum dilution 1:1000. A total of 8 replicate measurements were obtained for each *S. aureus* antigen. Also, let *MFI_ird_* denote the MFI of the secondary human IgG antibodies diluted by a factor *d=1,...,D*. Secondary IgG MFIs were measured at each of eight dilution points and for each subject over 12 replicates. Calibration consisted in: i) fitting a dilution-MFI curve to the secondary IgG MFIs of each subject and b) mapping the MFIs measured against the *S. aureus* antigens onto their fitted subject-specific secondary IgG concentration. Dilution-response curves of different subjects can be different, due to the combination of technical factors including the characteristics of secondary IgGs used to measure different subjects as well as the variation in performance of the photo-detectors over time. Mapping of each antigen’s MFI onto its corresponding secondary IgG concentration normalized the mapped concentrations with respect to these sources of subject-to-subject variations.

The dilution-MFI model used for calibration was:

log_2_(MFI_ird_) ~ Normal(M_id_,SD_id_) , (1)

where Normal(M,SD) denotes a Gaussian random variable with mean M and standard deviation SD and the mean was specified using the 4-parameters Emax model

M_id_ = α_i_ + (β_i_ -α _i_)/(1+e^(γi-log2(d))/δi^), (2)

where the subject-specific calibration parameters define the lower and upper asymptotes (α_i_ and β_i_ respectively), mid-point (γ_i_) and slope (δ_i_) of the mean MFI function. The standard deviation was defined as being proportional to the derivative of the mean function with respect to its dilution factor

SD_id_ = h_i_ ∂ M_id_/∂d, (3)

reflecting a serum-specific reduction in MFI variability due to assay saturation. Numerical maximum likelihood estimates of the calibration model parameters (α_i_, β_i_, γ_i_, δ_i_, h_i_) were calculated by letting the 12 replicate measurements at each dilution be conditionally independent random variables. Figure S.1 illustrates the results of this calibration process using the measured anti-human IgG MFIs for two illustrative subjects. MFI data are depicted in black in Figure 1, showing more variability away from the asymptotes of the calibration curve consistent with (3). Dashed lines in Figure S.1 delimit the point-wise 95% probability bands around the central estimate of the mean function.


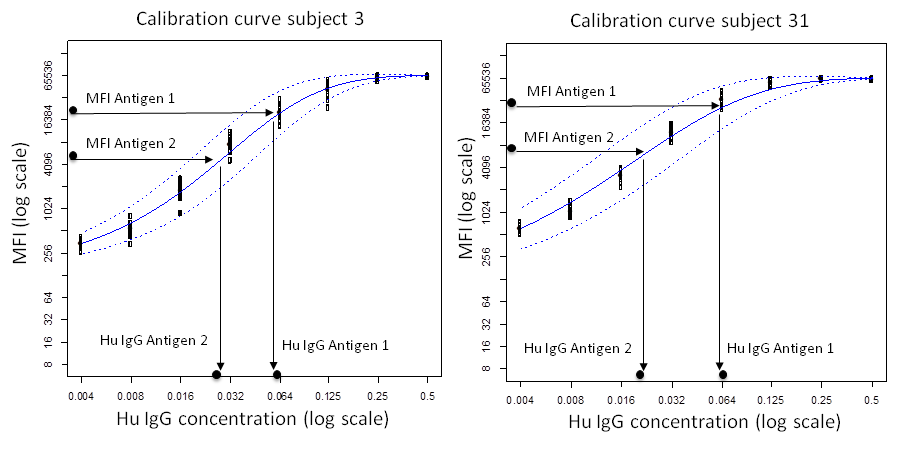


**Figure S.1:** Calibration of raw MFI data through mapping onto their corresponding human IgG secondary antibody concentration for the two illustrative study subjects 3 and 31.

*Dimensionality reduction*

Let c_ijr_ = M_id_^-1^(MFI_ijr_ | α_i_, β_i_, γ_i_, δ_i_) be the calibrated IgG level of replicate *r=1,...,R* MFI for subject *i=1,...,I* against antigen *j=1,...,J*. The superscript “-1” here is used to denote the inverse of the mean calibration function (2). Dimensionality reduction consisted in mapping the values (c_ij1_,...,c_ij8_) into one value c_ij_ of the IgG level for subject i against antigen j using the median of the eight replicate IgG levels. Median levels were used due to their robustness to outlying values compared to mean estimates.

*Background filtering*

Calibration and dimensionality reduction were performed both for the MFI measurements taken from the centre of each protein array spot and from its outer ring. Calibrated outer ring IgG levels were then used to assess the level of background noise affecting the measurements taken from the centre of the array spots. To this end, for each antigen, if an IgG level was smaller or equal to the 95^th^ percentile of the background distribution (depicted in Figure S.2) it was excluded from further analysis.


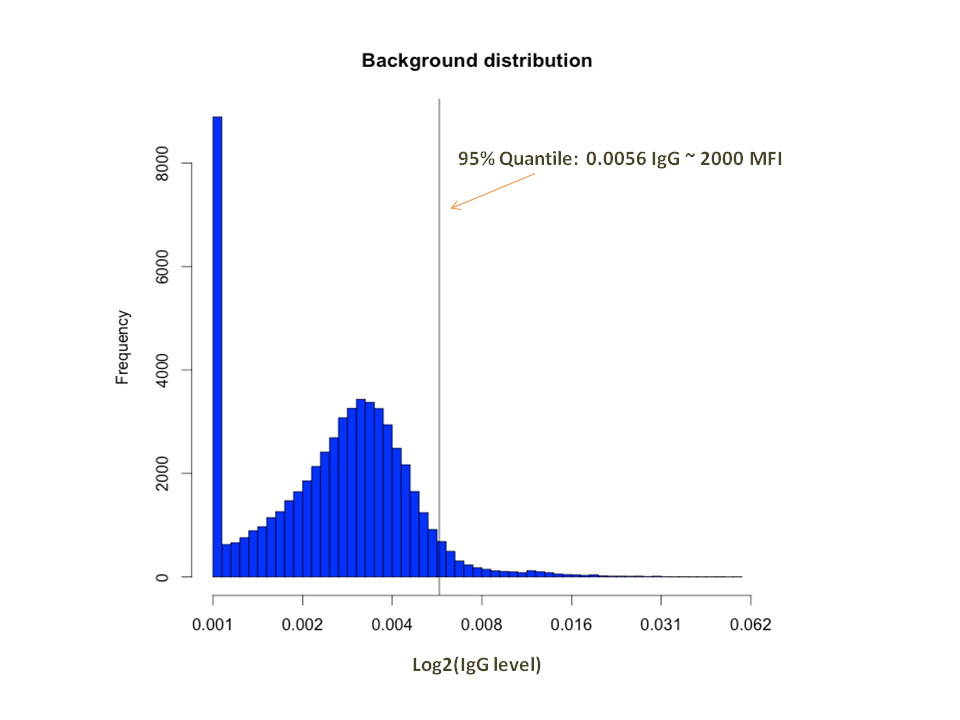


**Figure S.2:** Frequency distribution of the background IgG levels measured from the outer ring of each protein array spot. The background filtering level of 0.0056 μg/mL applied to the data was the estimated 95% quantile of this background distribution


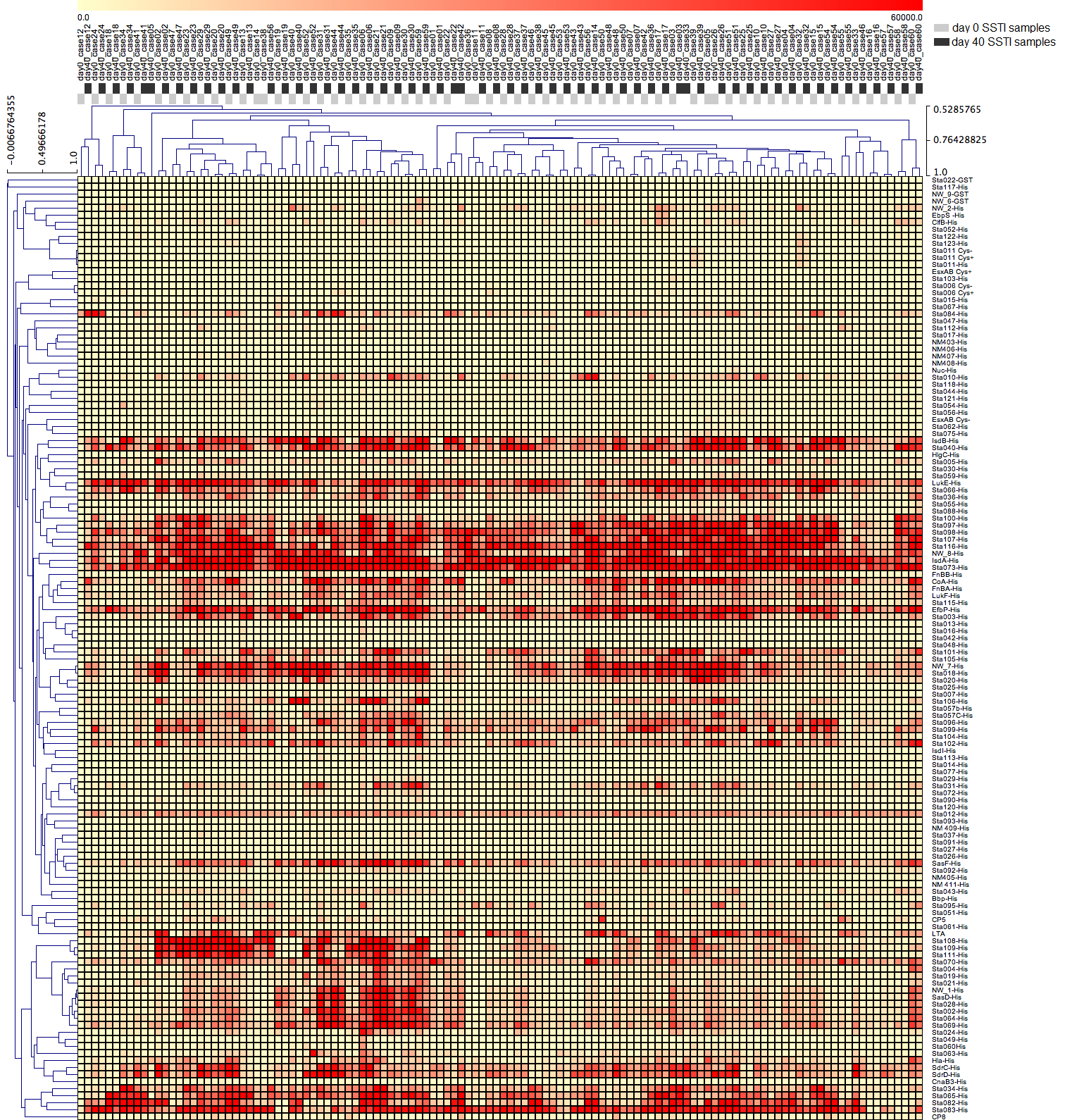


**Figure S.3:** Agglomerative clustering of the 60 SSTI samples at D0 (grey at the top of this figure) and D40 (black at the top of this figure) showing that the MFI data measured across all antigens in samples pertaining to the same subject were more similar to each other compared to the MFIs measured from different individuals for 53/60 ≈ 88% subjects (dendrogram at top of the figure). Also, horizontal red bands showed reactivity of the SSTI case sera with many of the 134 *S. aureus* antigens on the MFI scale

**Table S.1:** Antigens presented in Figure 3, indicating serum groups (case D0 and D40, and controls) differing in IgG levels and for each antigen, the locus tag, product name, and location in bacterial cell

| **Association** | **Locus_tag** | **Protein Product** | **Localization** |
| --- | --- | --- | --- |
| D0-Controls | HlgC (SAOUHSC_02709 | Gamma-hemolysin component C (Leukocidin s subunit) | SUBCELLULAR LOCATION: Secreted {ECO:0000269\|PubMed:20472795}. |
| D0-Controls | LTA |  |  |
| D0-Controls | Nuc  (SAOUHSC_01316) | Uncharacterized protein |  |
| D0-Controls | SAOUHSC_00661 | Uncharacterized protein |  |
| D0-Controls | SAOUHSC_01085 | High-affinity heme uptake system protein IsdE (Iron-regulated surface determinant protein E) (Staphylococcal iron-regulated protein F) | SUBCELLULAR LOCATION: Cell membrane {ECO:0000255\|PROSITE-ProRule:PRU00303}; Lipid-anchor {ECO:0000255\|PROSITE-ProRule:PRU00303}. |
| D0-Controls | SAOUHSC_01920 | Uncharacterized protein | Unknown |
| D0-Controls | SAOUHSC_00391 | Uncharacterized protein | Unknown |
| D0-Controls | SAOUHSC_00354 | Uncharacterized protein | Unknown |
| D0-Controls & D0-D40 | SAOUHSC_01127 | Uncharacterized protein | Unknown |
| D0-Controls & D0-D40 | SAOUHSC_00808 | Uncharacterized protein | Unknown |
| D0-Controls & D0-D40 | SAOUHSC_00390 | Uncharacterized protein | Unknown |
| D0-D40 | Coa  (SAOUHSC_00192) | Coagulase | Unknown |
| D0-D40 | FnBB  (SAOUHSC_02802) | Fibronectin binding protein B, putative | SUBCELLULAR LOCATION: Secreted, cell wall {ECO:0000256\|SAAS:SAAS00753613}; Peptidoglycan-anchor {ECO:0000256\|SAAS:SAAS00753613}. |
| D0-D40 | Csa3F  (NWMN_0408) | Staphylococcal tandem lipoprotein | Unknown |
| D0-D40 | SasF  (SAOUHSC_02982) | Uncharacterized protein | Unknown |
| D0-D40 | SAOUHSC_00171 | Gamma-glutamyltranspeptidase, putative (EC 2.3.2.2) | Unknown |
| D0-D40 | Csa3B  (SAOUHSC_00404) | Uncharacterized lipoprotein SAOUHSC_00404 | SUBCELLULAR LOCATION: Cell membrane {ECO:0000255\|PROSITE-ProRule:PRU00303}; Lipid-anchor {ECO:0000255\|PROSITE-ProRule:PRU00303}. |
| D0-D40 | SAOUHSC_02463 | Hyaluronate lyase (EC 4.2.2.1) (Hyaluronidase) (HYase) | SUBCELLULAR LOCATION: Secreted. |
| D0-D40 | SAOUHSC_00395 | Uncharacterized protein | Unknown |
| D0-D40 & D40-Controls | LukF  (SAOUHSC_02241) | Uncharacterized leukocidin-like protein 1 | SUBCELLULAR LOCATION: Secreted {ECO:0000269\|PubMed:20472795}. |
| D0-D40 & D40-Controls | Csa1B  (SAOUHSC_00053) | Uncharacterized lipoprotein SAOUHSC_00053 | SUBCELLULAR LOCATION: Cell membrane {ECO:0000255\|PROSITE-ProRule:PRU00303}; Lipid-anchor {ECO:0000255\|PROSITE-ProRule:PRU00303}. |
| D40-Controls | IsdB  (SAOUHSC_01079) | Iron-regulated surface determinant protein B (Fur-regulated protein B) (Staphylococcal iron-regulated protein H) (Staphylococcus aureus surface protein J) | SUBCELLULAR LOCATION: Secreted, cell wall {ECO:0000255\|PROSITE-ProRule:PRU00477}; Peptidoglycan-anchor {ECO:0000255\|PROSITE-ProRule:PRU00477}. |
| D40-Controls | SAOUHSC_00170 | Uncharacterized protein | Unknown |
| D40-Controls | SAOUHSC_01972 | Foldase protein PrsA (EC 5.2.1.8) | SUBCELLULAR LOCATION: Cell membrane {ECO:0000255\|HAMAP-Rule:MF_01145}; Lipid-anchor {ECO:0000255\|HAMAP-Rule:MF_01145}. |

HP, hypothetical protein

*D0 IgG<controls IgG; #D0 IgG<D40 IgG; $D40 IgG<controls IgG

**Summary Table S.2**: list of antigens whose IgG levels decreased the SSTI probabilities estimated by logistic regression when comparing D0-controls and D40-controls data, presented in rank order

| **Antigen IDs** | | **Protein Product** | **Localization** |
| --- | --- | --- | --- |
| **all 60 SSTI cases** | **30 culture-confirmed *S. aureus* SSTI cases** |  |  |
| SAOUHSC_00107 |  | Uncharacterized protein | Unknown |
| Csa1D  (SAOUHSC_00055) |  | Uncharacterized lipoprotein SAOUHSC_00055 | SUBCELLULAR LOCATION: Cell membrane {ECO:0000255\|PROSITE-ProRule:PRU00303}; Lipid-anchor {ECO:0000255\|PROSITE-ProRule:PRU00303}. |
| SAOUHSC_00872 |  | Extramembranal protein | Unknown |
| SAOUHSC_02767 | SAOUHSC_02767 | Peptide ABC transporter, peptide-binding protein, putative | Unknown |
| SAOUHSC_02554 |  | Uncharacterized protein | Unknown |
| SAOUHSC_00808 |  | Uncharacterized protein | Unknown |
| SAOUHSC_01854 |  | Uncharacterized protein | Unknown |
| SAOUHSC_00137 |  | Uncharacterized protein | Unknown |
| CnaB3  (NWMN_0525) |  | Serine-aspartate repeat-containing protein E | SUBCELLULAR LOCATION: Secreted, cell wall {ECO:0000255\|PROSITE-ProRule:PRU00477}; Peptidoglycan-anchor {ECO:0000255\|PROSITE-ProRule:PRU00477}. |
| SAOUHSC_00817 |  | Uncharacterized protein |  |
| SAOUHSC_01150 | SAOUHSC_01150 | Cell division protein FtsZ | SUBCELLULAR LOCATION: Cytoplasm {ECO:0000255\|HAMAP-Rule:MF_00909}. Note=Assembles at midcell at the inner surface of the cytoplasmic membrane. {ECO:0000255\|HAMAP-Rule:MF_00909}. |
| Csa3G  (NWMN_0409) | Csa3G  (NWMN_0409) | Staphylococcal tandem lipoprotein | Unknown |
| SAOUHSC_01084 |  | Uncharacterized protein | Unknown |
| SAOUHSC_02708 |  | Gamma-hemolysin h-gamma-ii subunit, putative | Unknown |
| SAOUHSC_00389 |  | Uncharacterized protein | Unknown |
| SAOUHSC_01112 | SAOUHSC_01112 | FPRL1 inhibitory protein (FLIPr) | SUBCELLULAR LOCATION: Secreted {ECO:0000250}. |
| NWMN_1877 |  | Chemotaxis inhibitory protein (CHIPS) | SUBCELLULAR LOCATION: Secreted {ECO:0000269\|PubMed:14993252}. |
| IsdI  (SAOUHSC_00130) |  | Heme oxygenase (staphylobilin-producing) 2 (EC 1.14.99.48) (Heme-degrading monooxygenase 2) (Iron-regulated surface determinant 2) (Iron-responsive surface determinant 2) | SUBCELLULAR LOCATION: Cytoplasm {ECO:0000255\|HAMAP-Rule:MF_01272}. |
| NWMN_0757 | NWMN_0757 | Secreted von Willebrand factor-binding protein | Unknown |
| SAOUHSC_02979 |  | N-acetylmuramoyl-L-alanine amidase domain-containing protein SAOUHSC_02979 | SUBCELLULAR LOCATION: Secreted {ECO:0000269\|PubMed:18621893, ECO:0000269\|PubMed:20472795}. |
| Nuc  (SAOUHSC_01316) |  | Uncharacterized protein |  |
| SAOUHSC_00391 |  | Uncharacterized protein |  |
| Csa1A  (SAOUHSC_00052) |  | Uncharacterized lipoprotein SAOUHSC_00052 | SUBCELLULAR LOCATION: Cell membrane {ECO:0000255\|PROSITE-ProRule:PRU00303}; Lipid-anchor {ECO:0000255\|PROSITE-ProRule:PRU00303}. |
| SAOUHSC_00248 | SAOUHSC_00248 | Glycyl-glycine endopeptidase LytM (EC 3.4.24.75) (Autolysin LytM) | SUBCELLULAR LOCATION: Secreted {ECO:0000269\|PubMed:10220159, ECO:0000269\|PubMed:20472795}. |
| SdrC  (SAOUHSC_00544) |  | Serine-aspartate repeat-containing protein C | SUBCELLULAR LOCATION: Secreted, cell wall {ECO:0000255\|PROSITE-ProRule:PRU00477}; Peptidoglycan-anchor {ECO:0000255\|PROSITE-ProRule:PRU00477}. |
| IsdA  (SAOUHSC_01081) |  | Iron-regulated surface determinant protein A (Fur-regulated protein A) (Staphylococcal transferrin-binding protein A) | SUBCELLULAR LOCATION: Secreted, cell wall {ECO:0000305\|PubMed:11952908}; Peptidoglycan-anchor {ECO:0000305\|PubMed:11952908}. |
| Csa3I  (NWMN_0411) | Csa3I  (NWMN_0411) | Staphylococcal tandem lipoprotein | Unknown |
| Csa2A  (SAOUHSC_00172) | Csa2A  (SAOUHSC_00172) | Uncharacterized protein SAOUHSC_00172 | SUBCELLULAR LOCATION: Cell membrane {ECO:0000305}; Single-pass membrane protein {ECO:0000305}. |
| Csa3C(  NWMN_0405) | Csa3C  (NWMN_0405) | Truncated staphylococcal tandem lipoprotein | Unknown |
| SAOUHSC_01180 | SAOUHSC_01180 | Uncharacterized protein | Unknown |
| SAOUHSC_02571 | SAOUHSC_02571 | Staphylococcal secretory antigen ssaA2 | SUBCELLULAR LOCATION: Secreted {ECO:0000269\|PubMed:20472795}. |
| SAOUHSC_00137 | SAOUHSC_00137 | Uncharacterized protein | Unknown |
| SAOUHSC_00106 | SAOUHSC_00106 | Uncharacterized protein | Unknown |

**Summary Table S.3**: list of 134 antigens belonging to the *S. aureus* strain NCTC 8325 or Newman, produced in *E. coli* as recombinant His6- or GST-tagged proteins.

| **Locus_tag** | **Product Name UniprotDB** | **Psort-B** | **Gi** | **Product Name** |
| --- | --- | --- | --- | --- |
| CnaB  (NWMN_0525) | Protein domain clones from Serine-aspartate repeat-containing (SdrE) | Cell wall | 151220737 | Ser-Asp rich fibrinogen/bone sialoprotein-binding protein SdrE |
| NWMN_0757 | secreted von Willebrand factor-binding protein | Extracellular | 151220969 | secreted von Willebrand factor-binding protein precursor |
| NWMN_0958 | Uncharacterized protein | Unknown | 151221170 | putative lipoprotein |
| NWMN_1876 | Staphylococcal complement inhibitor (SCIN) | Unknown | 151222088 | staphylococcal complement inhibitor SCIN |
| NWMN_1877 | Chemotaxis inhibitory protein (CHIPS) | Unknown | 151222089 | chemotaxis-inhibiting protein CHIPS |
| NWMN_1883 | Enterotoxin type A |  | 151222136 |  |
| NWMN_1924 | Uncharacterized protein |  | 151222095 |  |
| SAOUHSC_00051 | 1-phosphatidylinositol phosphodiesterase | Extracellular | 88193871 | 1-phosphatidylinositol phosphodiesterase precursor, putative |
| Csa1A  (SAOUHSC_00052) | Uncharacterized lipoprotein SAOUHSC_00052 | Unknown | 88193872 | putative lipoprotein |
| Csa1A  (SAOUHSC_00052) | Uncharacterized lipoprotein SAOUHSC_00052 | Unknown | 88193872 | putative lipoprotein |
| Csa1A  (SAOUHSC_00052) | Uncharacterized lipoprotein SAOUHSC_00052 | Unknown | 88193872 | putative lipoprotein |
| Csa1B  (SAOUHSC_00053) | Uncharacterized lipoprotein SAOUHSC_00053 | Unknown |  |  |
| Csa1C  (SAOUHSC_00054) | Uncharacterized lipoprotein SAOUHSC_00054 | Unknown |  |  |
| Csa1D  (SAOUHSC_00055) | Uncharacterized lipoprotein SAOUHSC_00055 | Unknown |  |  |
| SasD  (SAOUHSC_00094) | Uncharacterized protein SAOUHSC_00094 | Cell wall | 88193909 | SasD protein SAOUHSC_00094 |
| SAOUHSC_00106 | Uncharacterized protein | Unknown | 88193919 | hypothetical protein , leader |
| SAOUHSC_00107 | Uncharacterized protein | Extracellular | 88193920 | putative poly-gamma-glutamate capsule biosynthesis protein |
| IsdI  (SAOUHSC_00130) | Heme oxygenase (staphylobilin-producing) 2 | Unknown | 88193943 | heme-degrading monooxygenase IsdI |
| SAOUHSC_00137 | Uncharacterized protein | Unknown | 88193950 | putative lipoprotein |
| SAOUHSC_00170 | Uncharacterized protein | Unknown | 88193980 | extracellular solute-binding protein, RGD containing lipoprotein |
| SAOUHSC_00171 | gamma-glutamyltranspeptidase, putative | Extracellular | 88193981 | gamma-glutamyltranspeptidase, putative |
| Csa2A  (SAOUHSC_00172) | Uncharacterized protein SAOUHSC_00172 | Unknown | 88193982 | hypothetical protein SAOUHSC_00172 |
| SAOUHSC_00174 | M23/M37 peptidase domain protein | Extracellular | 88193984 | M23/M37 peptidase domain protein |
| SAOUHSC_00176 | Bacterial extracellular solute-binding protein, putative | Unknown | 88193986 | bacterial extracellular solute-binding protein, putative |
| SAOUHSC_00186 | lipoprotein, putative | Unknown | 88193996 | lipoprotein, putative |
| CoA  (SAOUHSC_00192) | coagulase | Extracellular | 88194002 | coagulase Coa |
| SAOUHSC_00201 | Uncharacterized protein | Cell wall | 88194011 | putative extracellular solute-binding protein |
| SAOUHSC_00248 | Glycyl-glycine endopeptidase LytM | Extracellular | 88194055 | peptidoglycan hydrolase, putative |
| SAOUHSC_00253 | Uncharacterized protein | Unknown | 88194059 | putative exported protein (hypo) |
| SAOUHSC_00256 | Uncharacterized protein | Unknown | 88194062 | secretory antigen SsaA-like protein |
| EsxA  (SAOUHSC_00257) | ESAT-6 secretion system extracellular protein A (Ess extracellular protein A) ; ESAT-6 secretion system extracellular protein B (Ess extracellular protein B) | Unknown | 88194063 | hypothetical protein SAOUHSC_00257 |
| EsxB  SAOUHSC_00265 | ESAT-6 secretion system extracellular protein A (Ess extracellular protein A) ; ESAT-6 secretion system extracellular protein B (Ess extracellular protein B) | Unknown | 88194070 | hypothetical protein SAOUHSC_00265 esxB |
| SAOUHSC_00279 | Uncharacterized protein | Unknown | 88194083 | putative lipoprotein |
| SAOUHSC_00300 | lipase 2 | Extracellular | 88194101 | lipase precursor |
| SAOUHSC_00354 | Uncharacterized protein | Unknown | 88194153 | staphylococcal enterotoxix, putative |
| SAOUHSC_00356 | Uncharacterized protein | Unknown | 88194155 | putative lipoprotein |
| SAOUHSC_00362 | Uncharacterized protein | Unknown | 88194160 | putative lipoprotein |
| SAOUHSC_00365 | Alkyl hydroperoxide reductase C | Cytoplasmic |  |  |
| SAOUHSC_00383 | Uncharacterized protein | Unknown | 88194180 | superantigen-like protein |
| SAOUHSC_00384 | Uncharacterized protein | Unknown | 88194181 | superantigen-like protein |
| SAOUHSC_00386 | Uncharacterized protein | Extracellular | 88194182 | superantigen-like protein |
| SAOUHSC_00389 | Uncharacterized protein | Extracellular | 88194184 | superantigen-like protein |
| SAOUHSC_00390 | Uncharacterized protein | Extracellular | 88194185 | superantigen-like protein 5 |
| SAOUHSC_00391 | Uncharacterized protein | Extracellular | 88194186 | superantigen-like protein |
| SAOUHSC_00392 | Uncharacterized protein | Extracellular | 88194187 | superantigen-like protein 7 |
| SAOUHSC_00393 | Uncharacterized protein | Extracellular | 88194188 | superantigen-like protein |
| SAOUHSC_00394 | Uncharacterized protein | Extracellular | 88194189 | superantigen-like protein |
| SAOUHSC_00395 | SAOUHSC_00817 | Extracellular | 88194190 | superantigen-like protein |
| SAOUHSC_00399 | Uncharacterized protein | Extracellular | 88194194 | superantigen-like protein |
| SAOUHSC_00400 | Uncharacterized protein | Unknown | 88194195 | putative surface protein (hypo) |
| Csa3B  (SAOUHSC_00404) | Uncharacterized lipoprotein SAOUHSC_00404 | Unknown | 88194198 | putative lipoprotein |
| SAOUHSC_00427 | N-acetylmuramoyl-L-alanine amidase sle1 | Cell wall | 88194219 | autolysin precursor, putative |
| SdrC  S(AOUHSC_00544) | Serine-aspartate repeat-containing protein C | Cell wall | 88194324 | sdrC protein, putative |
| SdrD  (SAOUHSC_00545) | Serine-aspartate repeat-containing protein D | Cell wall | 88194325 | sdrD protein, putative |
| SAOUHSC_00634 | ABC transporter, substrate-binding protein, putative | Unknown | 88194402 | ABC transporter, substrate-binding protein, putative \lipoprotein |
| SAOUHSC_00661 | Uncharacterized protein | Unknown | 88194426 | probable lipase |
| SAOUHSC_00671 | secretory antigen SsaA-like protein | Extracellular | 88194436 | secretory antigen SsaA-like protein |
| SAOUHSC_00685 | putative lipoprotein | Unknown | 88194450 | putative lipoprotein |
| SAOUHSC_00717 | Uncharacterized protein | Unknown | 88194482 | lipoprotein |
| SAOUHSC_00728 | Lipoteichoic acid synthase | Cytoplasmic Membrane | 88194493 | Predicted membrane-associated, metal-dependent hydrolase |
| SAOUHSC_00749 | Uncharacterized protein | Unknown | 88194514 | Siderophore binding protein FatB |
| SAOUHSC_00754 | Uncharacterized protein | Extracellular | 88194518 | ferrichrome binding protein |
| SAOUHSC_00754 | Uncharacterized protein | Unknown | 88194617 | 5-nucleotidase family protein |
| SAOUHSC_00773 | LysM domain protein | Unknown | 88194535 | immunogenic secreted precursor-like protein (truncated) |
| SAOUHSC_00808 | Uncharacterized protein | Unknown | 88194568 | putative lipoprotein |
| SAOUHSC_00817 | Uncharacterized protein | Cytoplasmic | 88194576 | von willebrand truncated |
| SAOUHSC_00872 | extramembranal protein | Cytoplasmic | 88194629 | extramembranal protein |
| SAOUHSC_00988 | Glutamyl endopeptidase | Extracellular | 88194745 | glutamyl endopeptidase precursor, putative |
| SAOUHSC_00994 | Bifunctional autolysin | Extracellular | 88194750 | bifunctional autolysin precursor, putative |
| SAOUHSC_01005 | Uncharacterized protein | Unknown | 88194760 | chitinase |
| IsdB  (SAOUHSC_01079) | Iron-regulated surface determinant protein B | Cell wall | 88194828 | neurofilament protein isdB |
| IsdA  (SAOUHSC_01081) | Iron-regulated surface determinant protein A | Cell wall | 88194829 | IsdA protein |
| SAOUHSC_01084 | Uncharacterized protein | Unknown | 88194831 | hypothetical iron-regulated protein, leader |
| SAOUHSC_01085 | High-affinity heme uptake system protein IsdE | Cytoplasmic | 88194832 | iron ABC transporter, iron -binding protein IsdE |
| SAOUHSC_01112 | FPRL1 inhibitory protein | Unknown | 88194858 | formyl peptide receptor-like 1 inhibitory protein |
| EfbP  (SAOUHSC_01114) | fibrinogen-binding protein | Cell wall | 88194860 | fibrinogen-binding protein truncated |
| SAOUHSC_01115 | Uncharacterized protein | Unknown | 88194861 | hypothetical protein SAOUHSC_01115 |
| Hla  (SAOUHSC_01121) | Alpha-hemolysin | Unknown | 88194865 | alpha-hemolysin precursor |
| SAOUHSC_01125 | Uncharacterized protein | Extracellular | 88194869 | superantigen-like protein |
| SAOUHSC_01127 | Uncharacterized protein | Extracellular | 88194870 | superantigen-like protein |
| SAOUHSC_01150 | cell division protein FtsZ | Cytoplasmic | 88194892 | cell division protein FtsZ |
| SAOUHSC_01180 | Uncharacterized protein | Unknown | 88194919 | putative lipoprotein |
| SAOUHSC_01219 | Probable cell wall hydrolase LytN | Cell wall | 88194955 | cell wall hydrolase, putative |
| SAOUHSC_01256 | Uncharacterized protein | Unknown | 88194989 | insulysin, peptidase family M16 |
| Nuc  (SAOUHSC_01316) | thermonuclease precursor /Uncharacterized protein | Extracellular | 88195046 | thermonuclease precursor |
| SAOUHSC_01317 | Uncharacterized protein | Unknown | 88195047 | hypothetical protein leader? |
| EbpS  (SAOUHSC_01501) | elastin binding protein EbpS | Cell wall | 88195217 | elastin binding protein EbpS |
| SAOUHSC_01508 | Uncharacterized protein | Unknown | 88195223 | putative lipoprotein |
| SAOUHSC_01854 | Uncharacterized protein | Cell wall | 88195552 | hypothetical protein SAOUHSC_01854 |
| SAOUHSC_01920 | Uncharacterized protein | Unknown | 88195615 | putative lipoprotein |
| SAOUHSC_01939 | serine protease SplC | Extracellular | 88195634 | serine protease SplC |
| SAOUHSC_01941 | serine protease SplB | Extracellular | 88195635 | serine protease SplB |
| SAOUHSC_01942 | serine protease SplA | Extracellular | 88195636 | serine protease SplA |
| SAOUHSC_01949 | intracellular serine protease, putative | Extracellular | 88195642 | intracellular serine protease, putative |
| LukE  (SAOUHSC_01955) | Leucotoxin LukEv | Extracellular | 88195648 | leukotoxin, LukE, putative |
| SAOUHSC_01972 | Foldase protein PrsA | Unknown | 88195663 | protein export protein PrsA, putative |
| SAOUHSC_02147 | Uncharacterized protein | Unknown | 88195827 | hypothetical protein, putative leader |
| SAOUHSC_02240 | Phospholipase C | Cell wall | 88195913 | truncated beta-hemolysin |
| LukF  (SAOUHSC_02241) | Uncharacterized leukocidin-like protein 1 | Extracellular | 88195914 | Leukocidin/Hemolysin toxin family LukF |
| SAOUHSC_02246 | Uncharacterized protein | Unknown | 88195918 | ferric hydroxamate receptor 1 |
| SAOUHSC_02333 | Probable transglycosylase SceD | Extracellular | 88195999 | Probable transglycosylase isaA precursor |
| SAOUHSC_02448 | Uncharacterized protein | Unknown | 88196100 | putative surface hydrolase |
| SAOUHSC_02463 | hyaluronate lyase | Extracellular | 88196115 | hyaluronate lyase |
| SAOUHSC_02554 | Uncharacterized protein | Unknown | 88196199 | ferrichrome-binding protein |
| SAOUHSC_02554 | Uncharacterized protein | Unknown | 88196199 | ferrichrome-binding protein |
| SAOUHSC_02571 | Staphylococcal secretory antigen ssaA2 | Extracellular | 88196215 | secretory antigen precursor, putative |
| SAOUHSC_02576 | secretory antigen precursor SsaA, putative | Unknown | 88196220 | secretory antigen precursor SsaA, putative |
| SAOUHSC_02576 | secretory antigen precursor SsaA, putative | Unknown | 88196220 | secretory antigen precursor SsaA, putative |
| SAOUHSC_02708 | gamma-hemolysin h-gamma-ii subunit, putative | Extracellular | 88196348 | gamma-hemolysin h-gamma-ii subunit, putative |
| HglC  (SAOUHSC_02709) | Gamma-hemolysin component C (Leukocidin s subunit) | Extracellular | 88196349 | leukocidin s subunit precursor, putative HlgC |
| SAOUHSC_02767 | Peptide ABC transporter, peptide-binding protein, putative | Cell wall | 88196403 | peptide ABC transporter, peptide-binding protein, putative |
| Csa4A  (SAOUHSC_02783) | Uncharacterized protein SAOUHSC_02783 | Unknown | 88196419 | hypothetical protein, putative leader |
| Csa4B  (SAOUHSC_02788) | Uncharacterized lipoprotein SAOUHSC_02788 | Unknown | 88196424 | hypothetical protein, putative leader |
| FnBB  (SAOUHSC_02802) | fibronectin binding protein B FnBPB | Cell wall | 88196437 | fibronectin binding protein B FnBPB |
| FnBA  (SAOUHSC_02803) | fibronectin-binding protein A FnBPA | Cell wall | 88196438 | fibronectin-binding protein A precursor FnBPA |
| SAOUHSC_02844 | Uncharacterized protein | Cytoplasmic | 88196477 | putative esterase, leader? |
| SAOUHSC_02883 | Staphylococcal secretory antigen SsaA | Extracellular | 88196512 | LysM domain protein |
| SAOUHSC_02887 | Probable transglycosylase IsaA | Unknown | 88196515 | immunodominant antigen A, putative |
| SAOUHSC_02963 | clumping factor B, putative | Cell wall | 88196585 | clumping factor B, putative |
| SAOUHSC_02971 | Aureolysin, putative | Extracellular | 88196592 | aureolysin, putative |
| SAOUHSC_02979 | N-acetylmuramoyl-L-alanine amidase domain-containing protein SAOUHSC_02979 | Extracellular | 88196599 | N-acetylmuramoyl-L-alanine amidase |
| SAOUHSC_02982 | Uncharacterized protein | Cell wall | 88196601 | sasF protein |
| SAOUHSC_03006 | Lipase 1 | Extracellular | 88196625 | lipase |
| Bbp | bone sialoprotein-binding protein | Extracellular |  |  |
| CP5 | Capsular polysaccharide type 5 |  |  |  |
| CP8 | Capsular polysaccharide type 8 |  |  |  |
| LTA | Catalog number ti tlrl-slta, Invivo, USA |  |  |  |
| Csa3A  (NWMN_0403) | Staphylococcal tandem lipoprotein | Unknown |  |  |
| Csa3C  (NWMN_0405) | Staphylococcal tandem lipoprotein | Unknown |  |  |
| Csa3D  (NWMN_0406) | Staphylococcal tandem lipoprotein | Unknown |  |  |
| Csa3E  (NWMN_0407) | Staphylococcal tandem lipoprotein | Unknown |  |  |
| Csa3F  (NWMN_0408) | Staphylococcal tandem lipoprotein | Unknown |  |  |
| Csa3G  (NWMN_0409) | Staphylococcal tandem lipoprotein | Cytoplasmic |  |  |
| Csa3I  (NWMN_0411) | Staphylococcal tandem lipoprotein | Unknown |  |  |

**Supplementary Data References**

Bagnoli F, Fontana MR, Soldaini E, Mishra RP, Fiaschi L, Cartocci E, Nardi-Dei V, Ruggiero P, Nosari S, De Falco MG, Lofano G, Marchi S, Galletti B, Mariotti P, Bacconi M, Torre A, Maccari S, Scarselli M, Rinaudo CD, Inoshima N, Savino S, Mori E, Rossi-Paccani S, Baudner B, Pallaoro M, Swennen E, Petracca R, Brettoni C, Liberatori S, Norais N, Monaci E, Bubeck Wardenburg J, Schneewind O, O'Hagan DT, Valiante NM, Bensi G, Bertholet S, De Gregorio E, Rappuoli R, Grandi G. Vaccine composition formulated with a novel TLR7-dependent adjuvant induces high and broad protection against *Staphylococcus aureus*. Proc Natl Acad Sci U S A. 2015 Mar 24;112(12):3680-5.

Moraschini L, Passalacqua I, Fabbrini M, Margarit Y Ros I, Rigat F. Threshold-free estimation of functional antibody titers of a group B streptococcus opsonophagocytic killing assay. Pharm Stat. 2015 May-Jun;14(3):189-97.

O'Connell DP, Nanavaty T, McDevitt D, Gurusiddappa S, Höök M, Foster TJ. The fibrinogen-binding MSCRAMM (clumping factor) of *Staphylococcus aureus* has a Ca2+-dependent inhibitory site. J Biol Chem. 1998 Mar 20;273(12):6821-9.

Roche FM, Massey R, Peacock SJ, Day NP, Visai L, Speziale P, Lam A, Pallen M, Foster TJ. Characterization of novel LPXTG-containing proteins of *Staphylococcus aureus* identified from genome sequences. Microbiology. 2003 Mar;149(Pt 3):643-54.

Scietti L, Sampieri K, Pinzuti I, Bartolini E, Benucci B, Liguori A, Haag AF, Lo Surdo P, Pansegrau W, Nardi-Dei V, Santini L, Arora S, Leber X, Rindi S, Savino S, Costantino P, Maione D, Merola M, Speziale P, Bottomley MJ, Bagnoli F, Masignani V, Pizza M, Scharenberg M, Schlaeppi JM, Nissum M, Liberatori S. Exploring host-pathogen interactions through genome wide protein microarray analysis. Sci Rep. 2016 Jun 15;6:27996.
